# Serum levels of environmental pollutants is a risk factor for breast cancer in Inuit: a case control study

**DOI:** 10.1186/s12940-017-0269-6

**Published:** 2017-06-13

**Authors:** Maria Wielsøe, Peder Kern, Eva Cecilie Bonefeld-Jørgensen

**Affiliations:** 1 Centre for Arctic Health & Molecular Epidemiology, Department of Public Health, Aarhus University, Aarhus, Denmark; 2 Department of Gynecology and Obstetrics, Dronning Ingrid’s Hospital, Nuuk, Greenland; 3 Institute for Nursing and Health Science, University of Greenland, Nuuk, Greenland

**Keywords:** Breast cancer, Arctic, Greenland, Environmental exposure, Perfluoroalkyl acid (PFAA), Polychlorinated biphenyl (PCB), Organochlorine pesticide (OCP)

## Abstract

**Background:**

Environmental Persistent Organic Pollutants (POPs) can alter the hormone homeostasis by mimicking, interfering or blocking the function of hormones; moreover POPs are hypothesized to modify the risk of breast cancer. The association between POPs and breast cancer has been widely studied but the conclusions are inconsistent. The present study examined the associations between serum levels of POPs and breast cancer with focus on the highly exposed Greenlandic Inuit population.

**Methods:**

The study design was a case-control study of Inuit women from Greenland. The participants were asked to complete a questionnaire with information on reproductive history and lifestyle and to provide a blood sample. The sampling was carried out in two time periods (2000–2003 and 2011–2014). The serum levels were determined of 14 polychlorinated biphenyls (PCBs), 11 organochlorine pesticides (OCPs), 16 perfluoroalkyl acids (PFAAs), 1 polybrominated biphenyl (PBB), and 9 polybrominated diphenyl ethers (PBDEs). Independent samples t-test was used to compare differences between cases and controls and odds ratios (OR) adjusted for identified confounders were obtained using logistic regression.

**Results:**

The study population included 77 breast cancer cases and 84 controls. The majority of the measured compounds declined significantly from 2000 – 2003 to 2011–2014. However, for the perfluorinated carboxylic acids (PFCAs) an increase was observed. The serum levels were significantly higher in cases compared to controls for the majority of the compounds, and after adjusting for age the difference was maintained for ∑OCP, dichlorodiphenyldichloroethylene (p,p′-DDE), ∑PFAA, ∑perfluorinated sulfonic acids (PFSA), perfluorohexane sulfonate (PFHxS), and perfluorooctane sulfonate (PFOS). For the lipophilic POPs, high serum levels (middel/highest vs. lowest tertile) of ∑PCB, ∑estrgoenicPCB, PCB99, PCB138, PCB153, PCB170, PCB170, and PCB183 was associated with breast cancer risk; for the amphiphilic PFAAs, high serum levels of ∑PFAA, ∑PFCA, ∑PFSA, perfluorooctanoic acid (PFOA), perfluorononanoic acid (PFNA), perfluorodecanoic acid (PFDA), PFHxS, and PFOS were associated with breast cancer risk.

**Conclusion:**

Significant, positive associations between breast cancer risk and PCBs and PFAAs were observed. The associations indicate that environmental exposure to POPs can be a factor increasing the risk for breast cancer in Inuit women.

**Electronic supplementary material:**

The online version of this article (doi:10.1186/s12940-017-0269-6) contains supplementary material, which is available to authorized users.

## Background

Normal development, growth, and function of the mammary glands depend on a well functioning and balanced endocrine system. Thus, endocrine-related processes altering the hormone levels such as early age at menarche, nulliparity or delayed childbearing, late age at menopause, and use of hormone replacement therapy are established risk factors for breast cancer [1, 2]. Environmental pollutants disturbing the hormone homeostasis by mimicking, interfering or blocking the function of the hormones, have been hypothesized to modify breast cancer risk.

Environmental pollutants, such as polychlorinated biphenyls (PCBs), organochlorine pesticides (OCPs), and perfluorinated alkylated acids (PFAAs), are reported potential endocrine disrupters [3]. Despite indications from in vitro and animal studies, the scientific evidence of associations between endocrine disrupting environmental pollutants and breast cancer risk in humans is inconclusive. Several critically literature reviews on association between exposure to PCBs and breast cancer risk have been conducted. Reviewing the literature published from 1996 to 2006, Brody et al. [4] concluded that there was evidence to support an association between PCB levels and breast cancer, however only in combination with certain genetic polymorphisms. Golden and Kimbrough [5] reported in a review consistently negative findings for increased breast cancer mortality in occupational studies and concluded that “the weight of evidence does not support a causal association for PCBs and human cancer”. In 2013, the International Agency for Research on Cancer determined that PCBs were carcinogenic [6]. The two recent literature reviews in 2015 and 2016 [7, 8] including meta-analyses analyzing sums of PCBs in functional groups both found significant associations. The grouping of PCBs was previously proposed by Wolff et al. [9]. Zhang et al. [7] reported an association with anti-estrogenic and dioxin-like PCBs (Group2) and CYP1A1 and CYP2B inducing PCBs (Group3). However, in the prospective studies alone, Zhang et al. did not report any significant associations. Leng et al. [8] included both traditional case-control and nested case-control studies with biological samples collected before diagnosis and found an association with estrogenic PCBs (Group1) and CYP1A1 and CYP2B inducing PCBs (Group3). Exposure to the legacy lipophilic pollutants (PCBs and OCPs) has received most attention and only a few epidemiological studies have been published on breast cancer risk as a result of exposure to the amphiphilic PFAAs. Perfluorooctanesulfonic acid (PFOS), perfluorooctanesulfonamide (PFOSA), and ∑perfluorinated sulfonic acids (PFSAs) were reported to increase breast cancer risk in both a prospective and a cross-sectional case-control study [10, 11].

Existing studies on environmental exposure and breast cancer risk are mainly conducted in Europe and America where the majority of the population belongs to the Caucasian race and the exposure levels are relatively low. It has been indicated that susceptibility to environmental pollutants differs between different ethnic groups. In the meta-analysis by Zhang et al. (2015), geographic heterogeneity in the association of PCB groups with breast cancer risk was observed [7]. In the US elevated exposure-related breast cancer risk due to occupationally exposure to PCBs was seen in non-white workers, but not in white workers [12]. Polymorphisms in the P450 enzyme system may alter the susceptibility to environmental exposures and interactions between these factors have been linked with breast cancer risk [13–15]. Genetic polymorphism differences in the P450 genes was found between Greenlandic Inuit and Europeans and may account for some of the variability in breast cancer incidence [16].

With the possible ethnic differences in mind, additional attention on breast cancer risk in ethnic populations is needed. In the present study, we assessed the influence of environmental pollutants on breast cancer risk in the highly exposed Greenlandic Inuit women.

## Method

### Study population

We used a case-control design. Participants were recruited during 2000–2003 and 2011–2014. The sampling has been described earlier by Bonefeld-Jørgensen et al. [11] for the period 2000–2003 and by Wielsøe et al. [17] for the period 2011–2014.

Breast cancer cases were recruited at diagnosis at Dronning Ingrids Hospital in Nuuk, Greenland. The breast cancer diagnosis was confirmed by a positive histological sample. Controls for the participants recruited during 2000–2003 were selected from two cross-sectional studies on healthy persons with serum measurements on persistent organic pollutant (POP) in the same period [18, 19]. The controls included from Cote et al. [18] were from Nuuk and controls from Deutch et al. were geographically more widespread [19] and included to ensure that the controls represented the general population and the cases. The controls recruited during 2011–2014 were patients with non malign diagnoses at the Dronning Ingrids Hospital and frequency matched on age and geographical living area [17]. The majority of the hospital-controls were admitted at the Department of Orthopaedic Surgery, and others were admitted at the Department of Gynaecology and Obstetrics and diagnosed with non malignant abnormities in uterus, ovaries and breast, including cysts, metrorrhagia, menorrhagia, and cystocele.

All participants had to be of Greenland Inuit descent, defined as having more than two grandparents born in Greenland.

A total of 115 controls and 31 cases were enrolled during 2000–2003 and 66 cases and 62 controls were enrolled during 2011–2014. To optimise the similarity of the study populations from the two recruitment periods, the controls from 2000 to 2003 were reduced to one control per case. From the 115 enrolled controls we selected 31 controls based on age and geographical living area in Greenland.

At enrollment the participants completed an assisted lifestyle questionnaire and provided a blood sample. For cases the blood samples were obtained before any treatment was initiated.

### Demographical factors

Information about age, body mass index (BMI), smoking status, menopause status, number of full term pregnancies, and history of breastfeeding was obtained from questionnaires.

### Measurement of persistent organic pollutants in serum

Serum levels of lipophilic persistent organic pollutant (POP) from both recruitment periods were measures at Le Centre de Toxicologie du Québec (Sainte-Foy, Québec, Canada). The POP measurements for the 2000–2003 collection are described elsewhere [11]. The same method was used to analyze the samples from 2011 to 2014, but more compounds were included. In samples from both recruitment periods the following compounds were measured: Total serum lipid, 12 PCBs [PCB 99, 101, 105, 118, 128, 138, 153, 156, 170, 180, 183, 187], and 8 OCPs [dichlorodiphenyltrichloroethane (p,p′-DDT), dichlorodiphenyldichloroethylene (p,p′-DDE), mirex, β-hexachlorocyclohexane (β-HCH), hexachlorobenzene (HCB), cis- and trans-nonachlor, and oxychlordane]. Furthermore, in the samples from the 2011–2014 collection measurements of an extra two PCBs [PCB 28, 52], 10 flame retardants including one polybrominated biphenyl [PBB153] and nine polybrominated diphenyl ethers (PBDEs) [PBDE 15, 17, 25, 28, 33, 47, 99, 100, 153], and three extra OCPs [aldrin, α-, γ- chlordane] were included. If the value was below the detection limit, we used the detection limit divided by two. All determined lipophilic POPs were normalized to the total serum lipid content analyzed in the same sample and reported as μg/kg lipid. Detection limits and detection frequencies are reported in the Additional file 1.

PFAAs were determined in serum samples at Department of Environmental Science, Aarhus University and details of the method have been described previously [10, 20]. In both recruitment periods the serum level of seven perfluorinated carboxylic acids (PFCAs) [perfluoroheptanoic acid (PFHpA, C7), perfluorooctanoic acid (PFOA, C8), perfluorononanoic acid (PFNA, C9), perfluorodecanoic acid (PFDA, C10), perfluoroundecanoic acid (PFUnA, C11), perfluorododecanoic acid (PFDoA, C12), perfluorotridecanoic acid (PFTrA, C13)], two perfluorinated sulfonic acids (PFSAs) [perfluorohexane sulfonate (PFHxS, C6) and perfluorooctane sulfonate (PFOS, C8)], and one sulfonamide (perflurooctanesulfonamide, PFOSA, C8) were determined. The 2011–2014 recruitment period also included analyses of the following PFCAs and PFSAs [perfluorohexanoic acid (PFHxA, C6), perfluoro-n-pentanoic acid (PFPeA, C5), perfluorotetradecanoic acid (PFTeA, C14), perfluorobutane sulfonate (PFBS, C4), perfluoroheptane sulfonate (PFHpS, C7), and perfluorodecane sulfonate (PFDS, C10)]. If the value was below the detection limit, we used the detection limit divided by two. Detection limits and detection frequencies are reported in the Additional file 1.

### Cotinine measurement

The plasma cotinine level was measured using Calbiotech Direct ELISA kit (Calbiotech Inc., USA) in accordance with the manufacturer’s protocol. Briefly, 10 μL of standard or sample was pipetted into the well, 100 μL Enzyme Conjugate was added and the plate was shaken for 30 s followed by 60 min of incubation. The wells were washed six times with 300 μL distilled water after incubation before adding 100 μL substrate reagents. After 30 min of additional incubation, 100 μL stop solution was added and the absorbance was detected at 450 nm on an EL8000 Universal Microplate Reader (BIO-TEK INSTRUMENTS, INC). Samples with levels higher than 100 ng/mL were diluted and measured again to ensure that the measured level was within the range of the standard controls. The measurement was carried out in duplets.

### Statistics

We report data on single compounds detected in more than 50% of the samples and analyses were also performed for the summed concentration of the compound groups: 1) ∑PCB: PCB 99, 101, 105, 118, 128, 138, 153, 156, 170, 180, 183, 187, 2) ∑PCB group 1 (estrogenic PCBs): PCB 101, 187 3) ∑PCB group 2 (anti-estrogenic and dioxin-like PCBs): PCB 105, 118, 128, 138, 156, 170, 4) ∑PCB group 3 (CYP1A1 and CYP2B inducing PCBs): PCB 99, 153, 180, 183, 5) ∑DL-PCBs (dioxin-like PCBs): PCB 105, 118, 156, 6) ∑OCP: p,p′-DDT, p,p′-DDE, mirex, β-HCH, HCB, cis- and trans-nonachlor, and oxychlordane, 7) ∑PFCA: PFHpA, PFOA, PFNA, PFDA, PFUnA, PFDoA and PFTrA, 8) ∑PFSA: PFHxS, PFOS and PFOSA, and 9) ∑PFAA: ∑PFCA + ∑PFSA. For sum calculations only the compounds measured in both recruitment periods were included.

Independent samples t-test was used to compare the demographical factors (age, BMI, plasma cotinine, number of full term pregnancies) and POP levels between cases and controls, and between estrogen-receptor-negative (ER-) and estrogen-receptor-positive (ER+) cases. The distribution of the variables was checked by Q-Q plots and when non-normal distribution was found, data was ln-transformed to improve the normality.

Pearson’s chi^2^- test was used to test for distribution differences in age groups, BMI groups, self-reported smoking status, menopause status, and ever breastfeed (yes/no) between cases and controls.

ANCOVA was used for comparison of the variables between cases and controls with adjustment for age. The analyses were performed on ln-transformed data. The assumption of linear relationship between outcome and the covariates was tested by visual inspection of scatterplots. In the analyses there was checked for outliers, homogeneity of regression slopes and homoscedasticity and homogeneity of variances.

To estimate the odds ratios (OR), unconditional logistic regression models were used and estimates were obtained under adjustment for potential confounders. The exposure levels were both considered as continuous and categorical tertiled variables, based on the distribution among the controls. Confounding by the variables of interest was assessed using the change-in-estimate approach through backward elimination [21]. The change-in-estimate between the full logistic model and the full model without the variable of interest was calculated. If the change in odds ratio estimate was above 10%, the variable was considered a confounder and included in the final model. Potential confounders considered for the analysis included age, cotinine levels, parity coded as a continuous variables, BMI in groups, and breastfeeding coded as yes or no.

Correlations were calculated using Spearman’s rho.

All estimates are reported with 95% confidence intervals (95% CI). The statistical analyses were produced using IBM SPSS Statistics 22 and the statistical significance level was set to *p* ≤ 0.05.

## Results

The study population of the present study includes 77 breast cancer cases and 84 controls. In Table 1 the demographic and lifestyle characteristics for the participants are presented. Age differed slightly between cases and controls: median age was 52 years for cases and 50 years for controls. The age group distribution was, however, similar between cases and controls. Cases had a slightly lower BMI than controls of 26.3 and 27.7 kg/m^2^, respectively. However, the distribution between normal weight, overweight and obese was not significantly different between cases and controls (Table 1).

**Table 1:** Baseline table for demographic and reproductive factors

Parameters	Cases				Controls				*p-value*
	*N* (*n*)	median	mean	95% CI	*N* (*n*)	median	mean	95% CI	
Demographic factors									
Age (years)	77 (77)	52.0	53.8	50.7; 57.0	84 (84)	50.0	49.3	46.6; 51.9	**0.039** ^**a**^
≤50	34 (44.2%)				50 (59.5%)				0.183^b^
51–55	11 (14.3%)				6 (7.1%)				
56–59	7 (9.1%)				8 (9.5%)				
≥60	25 (32.5%)				20 (23.8%)				
BMI (kg/m^2^)	77 (48)	26.3	25.8	24.6; 27.0	84 (75)	27.7	27.6	26.5; 28.7	**0.041** ^**a**^
<25	18 (37.5%)				23 (37.5%)				0.128^b^
25–30	24 (50.0%)				31 (41.3%)				
>30	6 (12.5%)				21 (28.8%)				
Smoking status	77 (70)				84 (80)				
Never	6 (8.6%)				16 (20.0%)				0.132^b^
Former	18 (25.7%)				20 (25.0%)				
Current	46 (65.7%)				44 (55.0%)				
Plasma cotinine (ng/ml)	77 (74)	46.3	132.4	91.5; 173.3	84 (76)	40.5	117.4	84.3; 150.6	0.209^a^
Reproductive factors									
Menopausal status	77 (66)				84 (75)				
Premenopausal	24 (36.4%)				24 (32.0%)				0.585^b^
Postmenopausal	42 (63.6%)				51 (68.0%)				
Full term pregnancies	77 (60)	3.0	3.0	2.4; 3.5	84 (67)	3.0	3.1	2.6; 3.6	0.799^a^
Breastfed	77 (52)				84 (68)				
Ever breastfed (Yes)	46 (88.5%)				59 (86.8%)				0.781^b^

^a^Independent samples t-test on ln-transformed variables; ^b^Pearson’s chi^2^-test; N total number of subjects; n number of subjects with information on the corresponding parameter; 95% CI: 95% confidence interval; *p*-value: significance testing between cases and controls; Bold text: significant finding; BMI: body mass index

The self-reported smoking status and plasma cotinine level were similar in the two groups. Although, 20.0% of controls and 8.6% of cases reported that they had never been smokers. Self-reported smoking status was highly correlated with the measured cotinine level (r_*s*_ = 0.801, *p* < 0.001, data not shown). Reproductive factors (menopause status, parity, and breastfeeding) did not differ between cases and controls. For the 2010–2014 recruitment period age at menche and age at menopause were also registred and no difference between cases and control were observed (data no shown).

Of the measured lipophilic POPs, four PCBs (28, 55, 101, and 128), three OCPs (aldrin, α-, and γ- chlordane), and all flame retardants (PBB153, PBDE 15, 17, 25, 28, 33, 47, 99, 100, 153) were below the detection limit in more than 50% of the samples and excluded from the single compound analyses. Detection limits and frequencies are reported in Additional file 1. The sum of PBDE was not calculated and examined as five of the nine compounds (PBDE 15, 17, 25, 28, and 99) were not detected in any of the samples and the samples above detection limit were less than 10% for the remaining PBDEs.

The level of the tested lipophilic POPs (PCBs and OCPs) was significantly higher in cases than in controls except for PCB105, PCB156, p,p’DDT, and βHCH (Table 2). Upon adjustment for age the significant difference only persisted for ΣOCP and p,p’DDE (Table 2). There were significant, strong and positive correlations between the individual lipophilic POPs with all Spearman’s rho coefficients above 0.70 (data not shown).

**Table 2:** Serum levels of lipophilic POPs (μg/kg lipid) in breast cancer cases and controls

Parameters	% > DL	Cases				Controls				*p-value* ^*a*^	*Age adjusted p-value* ^*b*^
		*n*	Median	P25-P75	Min-Max	*n*	Median	P25-P75	Min-Max		
∑PCB		76	1467.34	549.82–2965.41	116.40–9288.90	84	1156.90	302.20–2170.81	43.60–6902.20	**0.011**	0.114
∑PCB Grp1		76	132.25	53.25–249.66	12.50–1103.00	84	103.50	32.88–182.76	3.90–470.29	**0.007**	0.696
∑PCB Grp2		76	481.05	178.34–903.06	37.00–2128.90	84	352.65	101.68–749.23	13.90–1763.53	**0.022**	0.211
∑PCB Grp3		76	815.66	324.75–1838.50	66.90–6057.00	84	655.13	179.01–1192.76	23.40–4789.00	**0.008**	0.084
∑DL-PCB		76	124.22	40.35–241.86	9.00–527.17	84	96.50	33.50–186.67	3.60–439.85	**0.047**	0.386
PCB99	96.9	76	56.57	28.00–106.97	3.50–315.22	84	53.00	14.75–82.94	2.00–182.21	**0.022**	0.161
PCB101	53.6	76	4.62	2.53–8.66	1.50–39.58	84	4.63	2.50–9.30	0.75–45.20	0.794	0.478
PCB105	92.1	76	10.40	3.93–23.36	1.00–80.43	84	5.53	3.05–19.79	0.50–48.38	0.175	0.797
PCB118	88.1	76	69.18	24.00–146.98	5.70–369.57	84	58.00	20.75–107.85	2.60–242.65	**0.039**	0.300
PCB138	100.0	76	212.53	100.00–452.31	20.00–970.00	84	190.75	47.00–377.74	7.00–990.59	**0.018**	0.169
PCB153	100.0	76	486.36	185.00–1075.00	40.00–3100.00	84	375.00	98.61–666.83	14.00–2400.00	**0.006**	0.070
PCB156	98.0	76	34.50	11.11–68.77	1.89–270.00	84	20.71	6.03–53.50	0.50–217.79	0.057	0.516
PCB170	100.0	76	84.02	33.23–211.20	5.85–900.00	84	73.00	18.25–150.00	2.80–650.00	**0.022**	0.227
PCB180	100.0	76	266.36	98.41–637.61	20.75–2800.00	84	200.59	56.50–411.95	6.90–2200.00	**0.009**	0.104
PCB183	98.0	76	24.00	12.21–52.37	2.40–130.00	84	22.53	7.32–38.22	0.50–91.76	**0.012**	0.111
PCB187	100.0	76	122.36	50.50–240.43	9.00–1100.00	84	95.50	28.96–167.77	1.90–463.85	**0.012**	0.111
∑OCP		76	1933.96	752.60–3785.71	160.70–9765.00	84	1529.79	438.70–2405.10	53.35–6334.00	**0.004**	**0.042**
Cis-Nonachlor	99.3	76	65.00	23.00–128.75	6.20–228.26	84	50.53	18.00–92.80	0.35–190.00	**0.008**	0.077
Trans-Nonachlor	99.3	76	355.56	130.39–827.21	35.00–1700.00	84	276.30	86.00–475.79	0.50–980.00	**0.005**	0.054
HCB	100.0	76	190.00	73.00–362.17	20.00–693.18	84	135.00	56.94–255.26	10.00–686.63	**0.030**	0.241
Mirex	94.7	76	25.00	11.15–74.92	1.89–420.00	84	22.65	6.08–47.79	0.50–150.00	**0.018**	0.171
Oxychlordane	100.0	76	200.00	62.75–456.03	12.45–1100.00	84	138.86	40.42–290.63	1.00–730.00	**0.010**	0.096
p,p’DDE	100.0	76	950.00	415.00–1949.57	76.00–5800.00	84	779.20	232.50–1144.87	33.00–4100.00	**0.002**	**0.024**
p,p’DDT	69.3	75	20.00	7.00–34.00	3.00–132.61	84	10.00	4.50–26.97	2.99–86.76	0.112	0.356
βHCH	99.3	76	31.00	10.25–48.43	1.00–150.00	84	18.50	8.03–38.83	1.00–83.66	0.056	0.408

% > DL: % of samples above decetion limet; n: number of observations per group; P25-P75: 25 percentile – 75 percentile; ^a^ *p*-value for the difference between cases and controls tested on ln-transformed variables with independent samples t-test; ^b^ *p*-value for the difference between cases and controls when adjusting for age, tested with ANCOVA test with age as a covariate factor; Bold text: significant finding; ∑PCB: PCB 99, 101, 105, 118, 128, 138, 153, 156, 170, 180, 183, 187; ∑PCB group 1 (estrogenic PCBs): PCB 101, 187; ∑PCB group 2 (anti-estrogenic and dioxin-like PCBs): PCB 105, 118, 128, 138, 156, 170; ∑PCB group 3 (CYP1A1 and CYP2B inducing PCBs): PCB 99, 153, 180, 183; ∑DL-PCBs (dioxin-like PCBs): PCB 105, 118, 156; ∑OCP: p,p′-DDT, p,p′-DDE, mirex, β-HCH, HCB, cis- and trans-nonachlor, and oxychlordane. The serum concentration of the POPs was reported in μg/kg lipid

For all of the lipophilic POPs, the levels had significantly decreased from 2000 - 2003 to 2011–2014 (Table 3). The breast cancer risk was significantly positively associated with the majority of the PCBs (Table 4). The associations were, however, weak in the analyses of the continuous variables. Whereas in the tertiled analyses a significantly increased risk was seen in the middle and/or highest tertile of ΣPCB, ΣPCBgrp2, PCB99, PCB138, PCB153, PCB170, and PCB183 compared to the lowest tertile (Table 4), and a dose-response tendency were seen for many of the compounds. Slightly increased odds ratios was also seen for several of the OCPs when analyzed as continuous exposure variables; however, in the tertiled analyses none of the estimates reached significance (Table 5).

**Table 3:** Serum levels of lipophilic POPs (μg/kg lipid) in cases and controls in the two recruitment periods

Parameters	2000–2003			2011–2014			*p-value* ^a^	*p-value* ^b^	*p-value* ^c^
	Median serum levels			Median serum levels			*all*	*cases*	*controls*
	All	Cases	Controls	All	Cases	Controls			
∑PCB	1995.18	2048.80	1985.94	798.50	1014.90	577.40	**<0.001**	0.266	**<0.001**
∑PCB Grp1	173.75	172.07	173.75	76.00	93.35	53.50	**<0.001**	0.280	**<0.001**
∑PCB Grp2	755.42	696.47	767.50	220.80	290.65	173.10	**<0.001**	0.097	**<0.001**
∑PCB Grp3	1062.77	1106.39	1062.68	515.00	628.50	354.00	**0.002**	0.400	**0.001**
∑DL-PCB	173.86	149.32	182.90	60.00	70.05	49.90	**<0.001**	**0.044**	**<0.001**
PCB99	78.65	70.28	81.36	31.00	38.50	23.00	**<0.001**	**0.019**	**<0.001**
PCB101	7.27	5.44	9.16	3.00	3.00	3.00	**<0.001**	**<0.001**	**0.012**
PCB105	19.17	16.59	22.29	5.60	7.95	5.00	**<0.001**	**0.013**	**<0.001**
PCB118	99.76	99.61	99.76	34.00	48.50	32.00	**<0.001**	**0.019**	**<0.001**
PCB138	378.43	357.47	385.24	110.00	165.00	79.00	**<0.001**	**0.041**	**<0.001**
PCB153	585.14	636.81	583.66	270.00	355.00	200.00	**0.002**	0.399	**0.001**
PCB156	50.00	46.13	56.63	15.00	22.50	14.00	**<0.001**	0.256	**<0.001**
PCB170	120.00	113.62	128.75	51.00	65.50	36.00	**0.005**	0.676	**0.001**
PCB180	353.61	349.44	353.61	170.00	195.00	120.00	**0.007**	0.591	**0.002**
PCB183	36.94	39.83	36.72	13.00	18.50	12.00	**<0.001**	0.061	**<0.001**
PCB187	162.05	166.19	161.95	73.00	88.00	51.00	**<0.001**	0.061	**<0.001**
∑OCP	2303.45	2531.58	2127.71	1013.00	1539.50	847.00	**<0.001**	0.062	**<0.001**
Cis-Nonachlor	79.29	94.30	77.84	40.00	46.50	29.00	**<0.001**	0.165	**0.001**
Trans-Nonachlor	405.42	509.67	343.07	200.00	270.00	150.00	**0.011**	0.606	**0.004**
HCB	257.01	265.04	257.01	110.00	130.00	85.00	**<0.001**	**0.008**	**<0.001**
Mirex	37.50	33.52	40.82	19.00	21.00	13.00	**0.009**	0.926	**0.010**
Oxychlordane	235.94	301.72	213.64	110.00	115.00	82.00	**0.005**	0.429	**0.003**
p,p’DDE	1129.46	1288.21	1039.19	550.00	680.00	420.00	**<0.001**	**0.018**	**<0.001**
p,p’DDT	29.51	31.54	25.00	9.50	10.00	8.00	**<0.001**	**<0.001**	**<0.001**
βHCH	40.63	42.26	38.96	15.00	17.50	12.00	**<0.001**	**0.004**	**<0.001**

The differences between the two recruitment periods were tested with independent samples t-test. ^a^The *p*-value form independent samples t-test on ln-transformed variables between all the participants; ^b^The *p*-value from independent samples t-test on ln-transformed variables between the cases; ^c^The *p*-value from independent samples t-test on ln-transformed variables between the controls; Bold text: significant finding; ∑PCB: PCB 99, 101, 105, 118, 128, 138, 153, 156, 170, 180, 183, 187; ∑PCB group 1 (estrogenic PCBs): PCB 101, 187; ∑PCB group 2 (anti-estrogenic and dioxin-like PCBs): PCB 105, 118, 128, 138, 156, 170; ∑PCB group 3 (CYP1A1 and CYP2B inducing PCBs): PCB 99, 153, 180, 183; ∑DL-PCBs (dioxin-like PCBs): PCB 105, 118, 156; ∑OCP: p,p′-DDT, p,p′-DDE, mirex, β-HCH, HCB, cis- and trans-nonachlor, and oxychlordane. The serum concentration of the lipophilic POPs was measured in μg/kg lipid

**Table 4:** Odds ratio of breast cancer risk associated with serum levels of PCBs

	Unadjusted	Adjusted			
	Continuous (μg/kg lipid)	Continuous (μg/kg lipid)	1st Tertile	2nd Tertile	3rd Tertile
∑PCB (OR (95% CI))	**1.00**	**1.00 (1.00; 1.00)**	1.00 (reference)	1.93 (0.84; 4.43)	**2.50 (1.11; 5.63)**
n (cases/controls)	**76/84**	**76/84**	14/28	27/28	**35/28**
*p*-value	**0.008**	**0.008**		0.122	**0.027**
∑PCB Grp1 (OR (95% CI))	**1.00**	**1.00 (1.00; 1.01)**	1.00 (reference)	1.35 (0.60; 3.06)	2.12 (0.97; 4.16)
n (cases/controls)	**76/84**	**76/84**	17/28	23/28	36/28
*p*-value	**0.006**	**0.006**		0.468	0.059
∑PCB Grp2 (OR (95% CI))	**1.00**	**1.00 (1.00; 1.00)**	1.00 (reference)	**2.28 (1.01; 5.18)**	2.14 (0.94; 4.88)
n (cases/controls)	**76/84**	**76/84**	14/28	**32/28**	30/28
*p*-value	**0.029**	**0.029**		**0.048**	0.069
∑PCB Grp3 (OR (95% CI))	**1.00**	**1.00 (1.00; 1.00)**	1.00 (reference)	1.63 (0.72; 3.67)	2.13 (0.96; 4.69)
n (cases/controls)	**76/84**	**76/84**	16/28	26/28	34/28
*p*-value	**0.006**	**0.006**		0.242	0.062
∑DL-PCB (OR (95% CI))	1.00	1.00 (1.00; 1.01)	1.00 (reference)	1.10 (0.50; 2.41)	1.52 (0.71; 3.26)
n (cases/controls)	76/84	76/84	21/28	23/28	32/28
*p*-value	0.057	0.057		0.821	0.277
PCB99 (OR (95% CI))	**1.01**	**1.01 (1.00; 1.01)**	1.00 (reference)	**2.85 (1.25; 6.47)**	2.00 (0.86; 4.67)
n (cases/controls)	**76/84**	**76/84**	13/28	**37/28**	26/28
*p*-value	**0.039**	**0.039**		**0.013**	0.109
PCB101 (OR (95% CI))	1.00	1.00 (0.96; 1.05)	1.00 (reference)	1.73 (0.80; 3.71)	1.26 (0.56; 2.81)
n (cases/controls)	76/84	76/84	19/28	34/28	23/27
*p*-value	0.902	0.902		0.161	0.580
PCB105 (OR (95% CI))	1.01	1.01 (0.99; 1.04)	1.00 (reference)	1.11 (0.51; 2.41)	1.54 (0.71; 3.33)
n (cases/controls)	76/84	76/84	21/28	25/30	30/26
*p*-value	0.217	0.217		0.790	0.274
PCB118 (OR (95% CI))	**1.01**	**1.01 (1.00; 1.01)**	1.00 (reference)	0.83 (0.37; 1.84)	1.48 (0.70; 3.11)
n (cases/controls)	**76/84**	**76/84**	23/28	19/28	34/28
*p*-value	**0.026**	**0.026**		0.641	0.304
PCB138 (OR (95% CI))	**1.00**	**1.00 (1.00; 1.00)**	1.00 (reference)	**2.83 (1.22; 6.57)**	**2.50 (1.07; 5.85)**
n (cases/controls)	**76/84**	**76/84**	12/28	**34/28**	**30/28**
*p*-value	**0.048**	**0.048**		**0.015**	**0.035**
PCB153 (OR (95% CI))	**1.00**	**1.00 (1.00; 1.00)**	1.00 (reference)	2.15 (0.93; 4.99)	**2.69 (1.18; 6.14)**
n (cases/controls)	**76/84**	**76/84**	13/28	28/28	**35/28**
*p*-value	**0.005**	**0.005**		0.074	**0.019**
PCB156 (OR (95% CI))	1.00	1.00 (1.00; 1.01)	1.00 (reference)	1.69 (0.75; 3.80)	2.06 (0.93; 4.56)
n (cases/controls)	76/84	76/84	16/28	27/28	33/28
*p*-value	0.250	0.250		0.206	0.074
PCB170 (OR (95% CI))	**1.00**	**1.00 (1.00; 1.01)**	1.00 (reference)	2.00 (0.87; 4.58)	**2.43 (1.08; 5.48)**
n (cases/controls)	**76/84**	**76/84**	14/28	28/28	**34/28**
*p*-value	**0.022**	**0.022**		0.101	**0.033**
PCB180 (OR (95% CI))	**1.00**	**1.00 (1.00; 1.00)**	1.00 (reference)	0.93 (0.41; 2.08)	1.65 (0.78; 3.47)
n (cases/controls)	**76/84**	**76/84**	22/29	19/27	35/28
*p*-value	**0.012**	**0.012**		0.855	0.189
PCB183 (OR (95% CI))	**1.02**	**1.02 (1.00; 1.03)**	1.00 (reference)	**2.46 (1.07; 5.65)**	**2.39 (1.04; 5.49)**
n (cases/controls)	**76/84**	**76/84**	13/28	**32/28**	**31/28**
*p*-value	**0.013**	**0.013**		**0.034**	**0.041**
PCB187 (OR (95% CI))	**1.00**	**1.00 (1.00; 1.01)**	1.00 (reference)	1.56 (0.69; 3.54)	2.19 (0.99; 4.82)
n (cases/controls)	**76/84**	**76/84**	16/28	25/28	35/28
*p*-value	**0.005**	**0.005**		0.285	0.052

n: number of observations per group; OR: odds ratio; 95% CI: 95% confidence interval; Unadjusted analysis: Only OR, and not 95% CI, were reported for unadjusted data; Adjusted: adjusted for confounders identified by change in estimate, following confounders were considered age, BMI, cotinine levels, parity, and breastfeeding; Bold text**:** significant finding, OR si﻿gnificantly different from 1; ∑PCB: PCB 99, 101, 105, 118, 128, 138, 153, 156, 170, 180, 183, 187; ∑PCB group 1 (estrogenic PCBs): PCB 101, 187; ∑PCB group 2 (anti-estrogenic and dioxin-like PCBs): PCB 105, 118, 128, 138, 156, 170; ∑PCB group 3 (CYP1A1 and CYP2B inducing PCBs): PCB 99, 153, 180, 183; ∑DL-PCBs (dioxin-like PCBs): PCB 105, 118, 156

**Table 5:** Odds ratio of breast cancer risk associated with serum levels of OCPs

	Unadjusted	Adjusted			
	Continuous (μg/kg lipid)	Continuous (μg/kg lipid)	1st Tertile	2nd Tertile	3rd Tertile
∑OCP (OR (95% CI))	**1.00**	**1.00 (1.00; 1.00)**	1.00 (reference)	1.05 (0.47; 2.39)	1.90 (0.88; 4.07)
n (cases/controls)	**75/84**	**75/84**	19/28	20/28	36/28
*p*-value	**0.002**	**0.002**		0.902	0.101
Cis-Nonachlor (OR (95% CI))	**1.01**	**1.01 (1.00; 1.01)**	1.00 (reference)	1.31 (0.58; 2.96)	2.07 (0.96; 4.47)
n (cases/controls)	**76/84**	**76/84**	18/29	22/27	36/28
*p*-value	**0.008**	**0.008**		0.512	0.063
Trans-Nonachlor (OR (95% CI))	**1.00**	**1.00 (1.00; 1.00)**	1.00 (reference)	1.00 (0.44; 2.25)	1.80 (0.84; 3.84)
n (cases/controls)	**76/84**	**76/84**	20/28	20/28	36/28
*p*-value	**0.002**	**0.002**		1.000	0.128
HCB (OR (95% CI))	1.00	1.00 (1.00; 1.00)	1.00 (reference)	0.86 (0.38; 1.94)	1.76 (0.83; 3.73)
n (cases/controls)	76/84	76/84	21/28	23/28	32/28
*p*-value	0.062	0.062		0.712	0.138
Mirex (OR (95% CI))	**1.01**	**1.01 (1.00; 1.02)**	1.00 (reference)	1.42 (0.65; 3.12)	1.58 (0.73; 3.44)
n (cases/controls)	**76/84**	**76/84**	19/28	27/28	30/28
*p*-value	**0.026**	**0.026**		0.381	0.250
Oxychlordane (OR (95% CI))	**1.00**	**1.00 (1.00; 1.00)**	1.00 (reference)	1.11 (0.49; 2.49)	1.90 (0.88; 4.07)
n (cases/controls)	**76/84**	**76/84**	19/28	21/28	36/28
*p*-value	**0.005**	**0.005**		0.809	0.101
p,p’DDE (OR (95% CI))	**1.00**	**1.00 (1.00; 1.00)**	1.00 (reference)	1.69 (0.75; 3.80)	2.06 (0.93; 4.56)
n (cases/controls)	**76/84**	**76/84**	16/28	27/28	33/28
*p*-value	**0.002**	**0.002**		0.206	0.074
p,p’DDT (OR (95% CI))	1.01	1.01 (1.00; 1.03)	1.00 (reference)	1.52 (0.67; 3.42)	2.07 (0.95; 4.52)
n (cases/controls)	75/84	75/84	17/29	24/27	34/28
*p*-value	0.074	0.074		0.315	0.067
βHCH(OR (95% CI))	1.01	1.01 (1.00; 1.03)	1.00 (reference)	1.11 (0.49; 2.49)	1.90 (0.88; 4.07)
n (cases/controls)	76/84	76/84	19/28	21/28	36/28
*p*-value	0.071	0.071		0.809	0.101

n: number of observations per group; OR: odds ratio; 95% CI: 95% confidence interval; Unadjusted analysis: Only OR, and not 95% CI, were reported for unadjusted data; Adjusted: adjusted for confounders identified by change in estimate, following confounders were considered age, BMI, cotinine levels, parity, and breastfeeding; Bold text: significant finding, OR significantly different from 1; ∑OCP: p,p′-DDT, p,p′-DDE, mirex, β-HCH, HCB, cis- and trans-nonachlor, and oxychlordane

Similar odds ratio estimates for the lipophilic POP were observed for the two recruitment periods separately (Additional file 2). However, except for p,p’DDE, only the estimates from the 2011–2014 data set were statistically significant, which may be due to a smaller sample size and thus lower statistical power in the 2000–2003 dataset. Even though the etiology of breast cancer might differ between premenopausal and postmenopausal cancer, we observed similar odds ratio estimates when stratifying on menopausal status (data not shown).

Seven of the measured PFAAs (PFPeA, PFHxA, PFTrA, PFTeA, PFBS, PFDS, and PFOSA) were below the detection limit in more than 50% of the samples and thus excluded from the analyses of single compounds. Detection limits and frequencies are reported in Additional file 1.

The cases had significantly higher serum levels of the tested PFAAs compared to the controls except for PFNA and PFDoA (Table 6). Upon adjustment for age, the difference was only significant for the ∑PFAA, ∑PFSA, PFHxS, and PFOS. Significant, positive intercorrelations were found between the individual levels of PFAAs (data not shown).

**Table 6:** Serum levels of PFAA (ng/ml) in breast cancer cases and controls

Parameters	% > DL	Cases				Controls				*p-value* ^*a*^	*age adjusted p-value* ^*b*^
		*n*	median	P25-P75	Min-max	*n*	median	P25-P75	Min-max		
∑PFAA		77	48.90	21.60–95.49	7.22–283.68	81	27.91	14.56–63.62	2.96–160.56	**0.001**	**0.020**
∑PFCA		77	8.55	5.66–17.72	2.20–72.93	81	6.97	3.51–17.06	0.96–49.71	**0.016**	0.225
PFHpA	72.1	77	0.11	0.05–0.30	0.03–1.55	81	0.08	0.05–0.18	0.03–0.59	**0.022**	0.170
PFOA	96.9	77	2.08	1.33–2.91	0.20–9.52	81	1.48	0.90–2.40	0.20–6.29	**0.009**	0.139
PFNA	96.3	77	3.28	1.45–4.97	0.30–38.60	81	1.83	0.76–4.63	0.25–12.50	0.055	0.530
PFDA	99.4	77	1.30	0.80–2.90	0.20–11.10	81	1.01	0.43–2.52	0.05–6.41	**0.015**	0.177
PFUnA	98.1	77	2.23	1.63–5.09	0.20–24.90	81	2.02	0.90–4.66	0.03–20.0	**0.026**	0.253
PFDoA	57.1	77	0.40	0.21–0.73	0.15–5.71	81	0.21	0.21–0.88	0.15–6.49	0.496	0.719
∑PFSA		77	38.10	14.80–68.11	5.02–211.00	81	19.91	10.02–44.35	2.00–142.26	**0.001**	**0.014**
PFHxS	98.8	77	2.52	0.96–4.07	0.19–23.40	81	1.14	0.64–2.91	0.16–13.90	**0.002**	**0.031**
PFOS	100.0	77	35.50	13.45–62.75	4.23–187.00	81	18.2	8.99–41.40	1.70–133.00	**0.001**	**0.015**

% > DL: % of samples above decetion limet; n: number of observations per group; P25-P75: 25 percentile – 75 percentile; ^a^ *p*-value for the difference between cases and controls tested on ln-transformed variables with independent samples t-test; ^b^ *p*-value for the difference between cases and controls when adjusting for age, tested with ANCOVA test with age as a covariate factor; Bold text: significant finding; ∑PFCA: PFHpA, PFOA, PFNA, PFDA, PFUnA, PFDoA and PFTrA; ∑PFSA: PFHxS, PFOS and PFOSA; ∑PFAA: ∑PFCA + ∑PFSA. The serum concentration of PFAA was measured in ng/ml

For the two recruitment periods, the levels of ∑PFCA and the individual PFCAs (expect for PFOA) were significantly increased in the period 2011–2014 compared to the first period (2000–2003). In contrast, the levels of both the ∑PFSA and the individual PFSAs decreased significantly over time between the two recruitment periods, 2000–2003 and 2011–2014 (Table 7). A notably larger decrease was observed in cases compared to controls and the difference among controls between the two recruitment periods was not statistically significant (Table 7).

**Table 7:** Serum levels of PFAA in cases and controls in the two recruitment periods

Parameters	2000–2003			2011–2014			*P-value* ^a^	*P-value* ^b^	*P-value* ^c^
	Median serum levels			Median serum levels			*all*	*cases*	*controls*
	All	Cases	Controls	All	Cases	Controls			
∑PFAA	49.11	55.30	25.65	27.91	31.18	27.91	0.159	0.007	0.669
∑PFCA	6.59	7.95	4.78	9.08	9.83	8.75	**0.001**	**0.051**	**0.003**
PFHpA	0.05	0.05	0.05	0.14	0.17	0.13	**0.006**	0.084	**0.012**
PFOA	2.27	2.50	1.62	1.49	1.68	1.45	0.419	0.074	0.558
PFNA	1.50	1.80	0.89	2.64	3.03	2.58	**<0.001**	**0.001**	**<0.001**
PFDA	0.96	1.20	0.49	1.34	1.34	1.34	**<0.001**	**0.022**	**0.001**
PFUnA	1.80	2.00	0.98	2.49	2.58	2.35	**0.001**	**0.029**	**0.003**
PFDoA	0.15	0.20	0.15	0.54	0.56	0.48	**<0.001**	**<0.001**	**<0.001**
∑PFSA	42.80	48.20	20.71	19.90	21.39	19.90	**0.010**	**<0.001**	0.818
PFHxS	2.84	3.50	1.38	1.11	1.47	1.08	**<0.001**	**<0.001**	0.158
PFOS	39.70	45.60	18.06	18.20	19.35	18.20	**0.009**	**<0.001**	0.796

The differences between the two recruitment periods were tested with independent samples t-test. ^a^The *p*-value form independent samples t-test on ln-transformed variables between all the participants; ^b^The *p*-value from independent samples t-test on ln-transformed variables between the cases; ^c^The *p*-value from independent samples t-test on ln-transformed variables between the controls; Bold text: significant finding; ∑PFCA: PFHpA, PFOA, PFNA, PFDA, PFUnA, PFDoA and PFTrA; ∑PFSA: PFHxS, PFOS and PFOSA; ∑PFAA: ∑PFCA + ∑PFSA. The serum concentration of PFAS was measured in ng/ml

The analyses with continuous PFAA variables showed a significant, positive association between ∑PFAA, PFOA, ∑PFSA, PFHxS, and PFOS and breast cancer risk (Table 8). In the tertiled analyses a dose-response was observed for most of the compounds and a significantly increased odds ratio for ∑PFAA, ∑PFCA PFOA, PFNA, PFDA, ∑PFSA, PFHxS, and PFOS was seen for the middle and/or highest tertile compared to the lowest (Table 8). Similar odds ratio estimates were observed for the two recruitment periods when analyzed separately (Additional file 3). We did not observe differences in the odds ratio estimates when stratifying on menopausal status (data not shown).

**Table 8:** Odds ratio of breast cancer risk associated with serum levels of PFAAs

	Unadjusted	Adjusted			
	Continuous (ng/ml serum)	Continuous (ng/ml serum)	1st Tertile	2nd Tertile	3rd Tertile
∑PFAA (OR (95% CI))	**1.01**	**1.01 (1.00; 1.02)**	1.00 (reference)	**4.7 (1.78; 12.49)**	**5.29 (2.01; 13.92)**
n (cases/controls)	**81/77**	**81/77**	7/27	**33/27**	**37/27**
*p*-value	**0.008**	**0.008**		**0.002**	**0.001**
∑PFCA (OR (95% CI))	1.03	1.03 (1.00; 1.06)	1.00 (reference)	**2.29 (1.00; 5.21)**	2.21 (0.97; 5.21)
n (cases/controls)	81/77	81/77	14/27	**32/27**	31/27
*p*-value	0.089	0.089		**0.049**	0.059
PFHpA (OR (95% CI))	**11.41**	6.98 (0.61; 80.0)	1.00 (reference)	1.13 (0.40; 3.20)	1.52 (0.54; 4.24)
n (cases/controls)	**81/77**	59/45	14/21	13/17	18/21
*p*-value	**0.020**	0.119		0.816	0.425
PFOA (OR (95% CI))	**1.26**	**1.26 (1.01; 1.58)**	1.00 (reference)	1.86 (0.80; 4.31)	**2.64 (1.17; 5.97)**
n (cases/controls)	**81/77**	**81/77**	14/27	26/27	**37/27**
*p*-value	**0.039**	**0.039**		0.149	**0.019**
PFNA (OR (95% CI))	1.07	1.07 (0.98; 1.17)	1.00 (reference)	**2.43 (1.07; 5.51)**	2.07 (0.90; 4.76)
n (cases/controls)	81/77	81/77	14/27	**34/27**	29/27
*p*-value	0.116	0.116		**0.034**	0.086
PFDA (OR (95% CI))	1.17	1.17 (0.97; 1.40)	1.00 (reference)	2.14 (0.94; 4.91)	**2.36 (1.04; 5.36)**
n (cases/controls)	81/77	81/77	14/27	30/27	**33/27**
*p*-value	0.094	0.094		0.072	**0.041**
PFUnA (OR (95% CI))	1.06	1.06 (0.97; 1.15)	1.00 (reference)	2.13 (0.95; 4.81)	2.00 (0.88; 4.53)
n (cases/controls)	81/77	81/77	15/27	32/27	30/27
*p*-value	0.207	0.207		0.068	0.097
PFDoA (OR (95% CI))	1.03	1.03 (1.01; 1.06)	1.00 (reference)	1.67 (0.72; 3.84)	0.93 (0.45; 1.91)
n (cases/controls)	81/77	81/77	36/41	19/13	22/27
*p*-value	0.447	0.447		0.232	0.839
∑PFSA (OR (95% CI))	**1.01**	**1.01 (1.00; 1.02)**	1.00 (reference)	**3.25 (1.25; 8.45)**	**5.38 (2.13; 13.54)**
n (cases/controls)	**81/77**	**81/77**	8/27	**26/27**	**43/27**
*p*-value	**0.005**	**0.005**		**0.016**	**<0.001**
PFHxS (OR (95% CI))	**1.16**	**1.16 (1.02; 1.32)**	1.00 (reference)	1.13 (0.48; 2.66)	**2.69 (1.23; 5.88)**
n (cases/controls)	**81/77**	**81/77**	16/27	18/27	**43/27**
*p*-value	**0.029**	**0.029**		0.788	**0.013**
PFOS (OR (95% CI))	**1.02**	**1.02 (1.01; 1.03)**	1.00 (reference)	**3.13 (1.20; 8.15)**	**5.50 (2.19; 13.84)**
n (cases/controls)	**81/77**	**81/77**	8/27	**25/27**	**44/27**
*p*-value	**0.005**	**0.005**		**0.020**	**<0.001**

n: number of observations per group; OR: odds ratio; 95% CI: 95% confidence interval; Unadjusted analysis: Only OR, and not 95% CI, were reported for unadjusted data; Adjusted: adjusted for confounders identified by change in estimate, following confounders were considered age, BMI, cotinine levels, parity, and breastfeeding; Bold text: significant finding, OR significantly different from 1; ∑PFCA: PFHpA, PFOA, PFNA, PFDA, PFUnA, PFDoA and PFTrA; ∑PFSA: PFHxS, PFOS and PFOSA; ∑PFAA: ∑PFCA + ∑PFSA

**Fig. 1:**
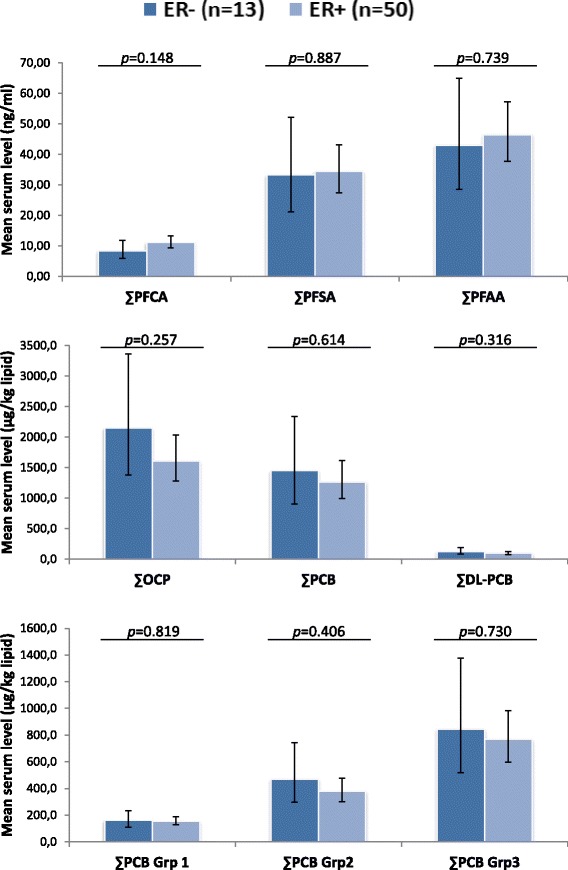
Serum levels of lipophilic POPs and PFAA in ER+ and ER- breast cancer cases. Mean serum POP estimates with 95% confidence interval (shown as *vertical lines*) obtained with ANCOVA analyses with age included as a covariate. The analyses were carried out on ln-transformed exposure variables and estimates were transformed back for the figure. *p*: *p*-value of the difference between ER+ and ER- cases; ∑PFCA: PFHpA, PFOA, PFNA, PFDA, PFUnA, PFDoA and PFTrA; ∑PFSA: PFHxS, PFOS and PFOSA; ∑PFAA: ∑PFCA + ∑PFSA; ∑PCB: PCB 99, 101, 105, 118, 128, 138, 153, 156, 170, 180, 183, 187; ∑DL-PCBs (dioxin-like PCBs): PCB 105, 118, 156; ∑OCP: p,p′-DDT, p,p′-DDE, mirex, β-HCH, HCB, cis- and trans-nonachlor, and oxychlordane; ∑PCB group 1 (estrogenic PCBs): PCB 101, 187; ∑PCB group 2 (anti-estrogenic and dioxin-like PCBs): PCB 105, 118, 128, 138, 156, 170; ∑PCB group 3 (CYP1A1 and CYP2B inducing PCBs): PCB 99, 153, 180, 183

Information on estrogen receptor status was available for 81.8% of the breast cancer cases. In the 63 cases with information on estrogen receptor status, 20.6% were ER- and 79.4% were ER+. The ∑OCP was significantly higher in ER- cases compared to ER+ cases (data not shown). When adjusting for age, there was no difference between ER- and ER+ cases (Fig. 1). None of the odds ratio estimates changed when the data was analyzed separately for the ER+ and ER- cases (data not shown).

**Fig. 2:**
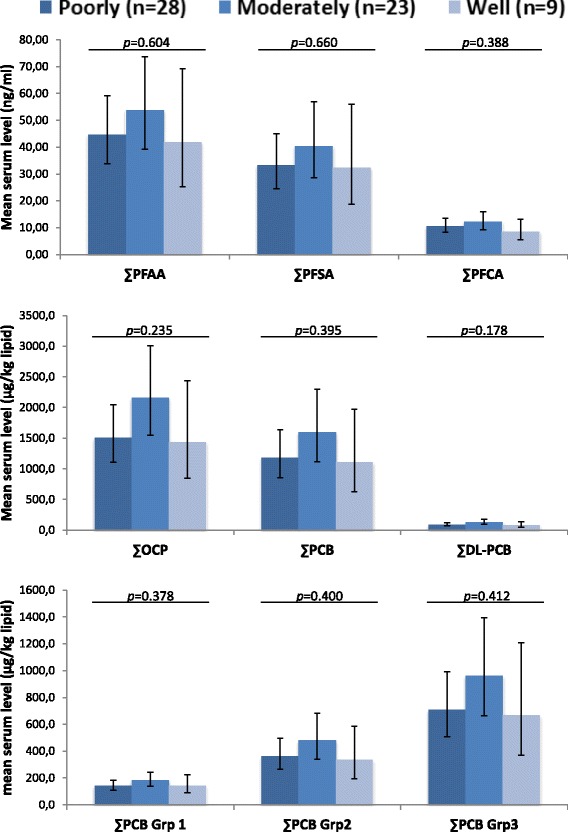
Serum levels of lipophilic POPs and PFAA in breast cancer cases with different differentiation grades. Mean serum POP estimates with 95% confidence interval (shown as *vertical lines*) obtained with ANCOVA analyses with age included as a covariate. The analyses were carried out on ln-transformed exposure variables and estimates were transformed back for the figure. *p*: *p*-value of the difference between ER+ and ER- cases; ∑PFCA: PFHpA, PFOA, PFNA, PFDA, PFUnA, PFDoA and PFTrA; ∑PFSA: PFHxS, PFOS and PFOSA; ∑PFAA: ∑PFCA + ∑PFSA; ∑PCB: PCB 99, 101, 105, 118, 128, 138, 153, 156, 170, 180, 183, 187; ∑DL-PCBs (dioxin-like PCBs): PCB 105, 118, 156; ∑OCP: p,p′-DDT, p,p′-DDE, mirex, β-HCH, HCB, cis- and trans-nonachlor, and oxychlordane; ∑PCB group 1 (estrogenic PCBs): PCB 101, 187; ∑PCB group 2 (anti-estrogenic and dioxin-like PCBs): PCB 105, 118, 128, 138, 156, 170; ∑PCB group 3 (CYP1A1 and CYP2B inducing PCBs): PCB 99, 153, 180, 183

Most tumors were classified as poorly (46.7%) or moderately (38.3%) differentiated. The serum levels of the tested compounds did not differ statistically between the tumor grades, however the levels were consistently highest among the cases with moderately differentiated tumors (Fig. 2).

## Discussion

The results of the present study suggest a positive association between breast cancer risk in Greenlandic Inuit women and the measured serum POP levels. We found that the breast cancer risk may be increased by exposure to PCBs and PFAAs, especially PFSA, and to a smaller extent by exposure to OCPs.

The Greenlandic Inuit population is exposed to high concentrations of lipophilic POPs through their traditional food, especially through intake of marine mammals [22–24]. Generally, the Arctic population displays a higher body burden of lipophilic POPs compared with the general populations in Europe and USA [23, 25]. Although also exposed through comsumer products, the main exposure source of PFAAs is intake of contaminated food [26]. A high correlation between serum PFAA and n-3/n-6 fatty acids in Greenland suggests that seafood may be an important exposure source in this population [27]. Both lipPOPs and PFAAs were also significantly, positive correlated with n-3/n-6 fatty acids in the present study (data not shown).

The association between exposure to environmental pollutants and breast cancer risk has been widely studied [4, 5, 7, 8, 10, 28, 29], however, the reported results is inconclusive. In vitro and animal studies report endocrine disrupting effects of numerous of the POPs investigated in the present study. Endocrine disrupting POPs could cause dysregulation of hormone signaling and cell function and thereby increase breast cancer risk. Estrogenic and anti-estrogenic effects of PCBs and complex lipPOP mixtures extracted from human serum were observed in a number of in vitro studies [30–35] while others found no effects [36]. Endocrine disrupting effects of PFAAs are demonstrated both in vitro*,* in animals studies and ex vivo studies of complex serum mixtures extracted from Danish pregnant women [37–40]. Studies in mice have concluded that gestational and chronic adult exposure to PFOA alters normal mammary development [39].

Other mechanisms of POP influencing breast cancer risk have been proposed, such as, promoting development of obesity ultimately leading to development and/or progression of breast cancer [41], disruption of the epigenomic landscape [42], induction of enzymes generating genotoxic intermediates [43], and induction of cytochrome 450 leading to increased levels of reactive oxygen or nitrogen species [44].

It is generally accepted that estrogen exposure alters the risk of breast cancer development and progression. The same mechanisms may alter breast cancer risk concerning POPs with estrogenic potential. However, when analyzing the PCBs by their potential mechanism of action as potentially estrogenic (Group 1), potentially antiestrogenic, immunotoxic, and dioxin-like (Group 2), and CYP1A and CYP2B inducers (Group 3) we did not observe any difference in odds ratios between the groups. The individual serum PCB levels were, however, strongly correlated and the similar odds ratio estimates may result from uncontrolled confounding that cannot be eliminated. For each of the three PCB groups we found an increased risk in the analyses with continuous exposure variables, although with odds ratios close to 1.00 (Table 4). The ∑PCBgrp2 was the only group significantly associated with breast cancer in the tertiled analyses, but a dose-response association was missing. Our results from the continuous analyses are in line with two meta-analyses reporting that all three groups of PCBs may increase breast cancer risk [7, 8]. The meta-analyses found a stronger association than observed in the present study, which might partly be explained by the lower statistical power in our study.

While several studies report on the association between breast cancer and PCBs, only a few have investigated associations with PFAAs. We found in a prospective Danish study an increased risk of the highest PFOSA quintile (Relative risk: 2.40 (1.20; 4.83)) and the association were strongest among the young women (below 40 years of age) [10]. In a previous case-control study in Greenland we found an association between breast cancer and PFOS (OR:1.03 (1.00; 1.07)) and ∑PFSA (OR:1.03 (1.00; 1.05)) [11] and in additional subsequent analyses we found that PFOSA increased breast cancer risk considerably (OR:6.13 (1.12–33.64)) [10]. It should be noted that the participants from the recruitment period 2000–2003 in the present study also participated in the previous Greenlandic study [11]. PFOSA has not been detected in any of the samples from the recruitment period 2011–2014, which may be due to regulations and a more rapid decrease of PFOSA serum levels. The same tendency in PFOSA serum levels has been observed in Denmark [45]. In both the Danish [10], the previous Greenlandic [11] and the present study the association between PFAA exposure and breast cancer was strongest for PFSAs, and weak or non-significant for PFCAs.

Polymorphisms in the cytochrome 450 system (CYP450) have been shown to interact with the effects of POPs on breast cancer risk [4, 13, 14, 46, 47]. The different results observed in studies investigating the effect of POP exposure on breast cancer risk may be explained by different allele frequencies of the polymorphisms among the populations. We have, previously reported that allele frequencies in CYP450 genes differ between European and the Greenlandic Inuit population, including the CYP1A1 Ile462Val (rs1048943) [16]. Furthermore, we have observed that the Val allele, which was more frequent in the Inuit population, increases risk of breast cancer in Greenland [15].

Breast cancer tumors can be classified as either ER- or ER+. ER- tumors are not influenced by estrogen and do not respond to hormone therapy treatment. ER+ tumors, respond to the hormone therapy and have a stronger association with estrogen-related factors such as early age at menarche, nulliparity or delayed childbearing, and postmenopausal obesity [48]. We did not observe any significant differences in serum POP levels between ER- and ER+ cases. Our findings are in line with Holmes et al. [29] reporting a non-significant higher level of OCPs, PCBs, and PBDEs in ER−/PR- cases compared to ER+/PR+ cases. A tendency to higher serum estrogen levels in patients with ER- tumors has been described in cases where blood samples were drawn after breast cancer development. The lower estrogen levels in ER+ might be explained by the ER+ tumors grown-dependent up-take of estrogen and thereby lowering the serum estrogen levels [49]. As our blood samples were drawn at breast cancer diagnosis, the estrogen receptor status may influence the estrogenic serum POP levels in the same way as estrogen. However, our results indicated that the receptor status did not influence the serum POP level. Furthermore, the estrogen receptor status did not seem to modify the effect of POP exposure on breast cancer risk, as the strata-specific measures of association were similar.

Our study has several limitations. The study design may cause selection bias, since selecting a comparable control group can be difficult. The cases were slightly older and leaner than the controls, although frequencies in age and BMI groups did not differ. The differences might influence our results but both age and BMI were considered to be potential confounders in the analyses. Furthermore, some of the controls were hospital patients with nonmalignant abnormalities in the uterus, ovaries and breasts. This may have caused a bias, most likely toward the null and then the effect may be greater than reported in the present study.

Our study determined the serum POP levels at the time of diagnosis. The body chemistry, metabolism etc. may have changed during disease development. The fact that the cases in our study were leaner than the controls may be due to disease-related weight loss, which is a symptom of advanced breast cancer. Weight loss has been reported to increase the lipid adjusted plasma concentrations of OCPs and PCBs [50, 51]. Information on recent weight loss was not available in our study, thus we were unable to adjust for this factor which might have influenced our results. The potential influence may be most pronounced for the lipophilic POPs accumulating in the adipose tissue compared to PFAA mainly concentrated in organs such as kidney, liver, brain and also blood. Furthermore, we used the serum measurements as indicator of breast tissue exposure. Preferably, the concentration should have been measured in the adipose tissue; however, studies have reported that serum levels of the lipophilic POPs are highly correlated with the level in breast tissue [52, 53]. Using serum POP levels appears to be a reasonable biomarker for long-term exposure. For PFAA the correlations have been less studied and the correlation between the matrices might be lower due to different physical characteristics.

## Conclusions

Our study showed a positive association between breast cancer risk and most of the measured POPs. Although, associations are weak, they indicate that exposure to environmental pollutants may be a risk factor for breast cancer in the Greenlandic Inuit women. A case-control study nested within the existing Greenlandic cohorts with prospectively colledted samples in more relevant timeframes is warranted to further evaluate the effect of POPs.

## Additional files

Additional file 1: Detection limits and frequencies of the measured compounds. (PDF 61 kb)

Additional file 2: Odds ratio of breast cancer risk associated with lipophilic POPs stratified for recruitment period. (PDF 133 kb)

Additional file 3: Odds ratio of breast cancer risk associated with PFAA stratified for recruitment period. (PDF 102 kb)

## Abbreviations

95% CI: 95% confidence interval
BMI: Body mass index
ER-: Estrogen-receptor-negative
ER+: Estrogen-receptor-positive
HCB: Hexachlorobenzene
OCP: Organochlorine pesticide
OR: Odds ratios
p,p′-DDE: Dichlorodiphenyldichloroethylene
p,p′-DDT: Dichlorodiphenyltrichloroethane
PBB: Polybrominated biphenyl
PBDE: Polybrominated diphenyl ether
PCB: Polychlorinated biphenyl
PFAA: Perfluorinated alkylated acid
PFBS: Perfluorobutane sulfonate
PFCA: Perfluorinated carboxylic acid
PFDA: Perfluorodecanoic acid
PFDoA: Perfluorododecanoic acid
PFDS: Perfluorodecane sulfonate
PFHpA: Perfluoroheptanoic acid
PFHpS: Perfluoroheptane sulfonate
PFHxA: Perfluorohexanoic acid
PFHxS: Perfluorohexane sulfonate
PFNA: Perfluorononanoic acid
PFOA: Perfluorooctanoic acid
PFOS: Perfluorooctanesulfonic acid
PFOSA: Perfluorooctanesulfonamide
PFPeA: Perfluoro-n-pentanoic acid
PFSA: Perfluorinated sulfonic acid
PFTeA: Perfluorotetradecanoic acid
PFTrA: Perfluorotridecanoic acid
PFUnA: Perfluoroundecanoic acid
POP: Persistent organic pollutant
β-HCH: β-hexachlorocyclohexane
