# Supplementary material for: Serum levels of environmental pollutants is a risk factor for breast cancer in Inuit: a case control study
Source: Environ Health. 2017 Jun 13;16:56. doi: 10.1186/s12940-017-0269-6 (PMC5470290; doi:10.1186/s12940-017-0269-6)
Supplement: Supplementary file 1 — Detection limits and frequencies of the measured compounds. (PDF 61 kb) [file 12940_2017_269_MOESM1_ESM.pdf]

Additional file 1

Serum levels of environmental pollutants is a risk factor for breast cancer in Inuit: A case control study

Maria Wielsøe, Peder Kern and Eva C. Bonefeld-Jørgensen

Limit of detection (LOD) of the measure compounds and the detection frequencies in the total samples set and the two collection periods separately

	**Total**	2000-2003		2011-2014	
Compound	**% Above LOD**	LOD	% Above LOD	LOD	% Above LOD
**PFBS***	**0.0**			0.02 ng/ml	0.0
**PFHxS**	**98.8**	0.4 ng/ml	96.8	0.03 ng/ml	100.0
**PFHpS***	**95.0**			0.04 ng/ml	95.0
**PFOS**	**100.0**	0.1 ng/ml	100.0	0.09 ng/ml	100.0
**PFDS***	**2.0**			0.12 ng/ml	2.0
**PFOSA**	**27.3**	0.2 ng/ml	69.4	0.40 ng/ml	1.0
**PFPeA***	**0.0**			0.06 ng/ml	0.0
**PFHxA***	**0.0**			0.01 ng/ml	0.0
**PFHpA**	**72.1**	0.1 ng/ml	51.6	0.02 ng/ml	84.9
**PFOA**	**96.9**	0.4 ng/ml	91.9	0.07 ng/ml	100.0
**PFNA**	**96.3**	0.5 ng/ml	90.3	0.09 ng/ml	100.0
**PFDA**	**99.4**	0.1 ng/ml	98.4	0.03 ng/ml	100.0
**PFUnA**	**98.1**	0.2 ng/ml	95.2	0.05 ng/ml	100.0
**PFDoA**	**57.1**	0.3 ng/ml	54.8	0.14 ng/ml	58.6
**PFTrA**	**35.4**	0.3 ng/ml	69.4	0.14 ng/ml	14.1
**PFTeA***	**0.0**			0.14 ng/ml	0.0
**Aldrin***	0.0			0.01 µg/L	0.0
**Cis-Nonachlor**	**99.3**	0.04 µg/L	100.0	0.005 µg/L	99.0
**Trans-Nonachlor**	**99.3**	0.04 µg/L	100.0	0.01 µg/L	99.0
**HCB**	**100.0**	0.04 µg/L	100.0	0.04 µg/L	100.0
**Mirex**	**94.7**	0.04 µg/L	100.0	0.01 µg/L	91.9
**Oxychlordane**	**100.0**	0.04 µg/L	100.0	0.005 µg/L	100.0
**alpha-Chlordane***	2.0			0.01 µg/L	2.0
**gamma-Chlordane***	0.0			0.005 µg/L	0.0
**p,p'-DDE**	**100.0**	0.08 µg/L	100.0	0.09 µg/L	100.0
**p,p'-DDT**	**69.3**	0.08 µg/L	90.6	0.05 µg/L	58.2
**ß-HCH**	**99.3**	0.08 µg/L	100.0	0.01 µg/L	99.0
**PBB153***	**17.2**			0.03 µg/L	17.2
**PBDE15***	**0.0**			0.03 µg/L	0.0
**PBDE17***	**0.0**			0.03 µg/L	0.0
**PBDE25***	**0.0**			0.03 µg/L	0.0
**PBDE28***	**0.0**			0.03 µg/L	0.0
**PBDE33***	**3.0**			0.03 µg/L	3.0

**PBDE47***	**7.1**			0.03 µg/L	7.1
**PBDE99***	**0.0**			0.02 µg/L	0.0
**PBDE100***	**1.0**			0.02 µg/L	1.0
**PBDE153***	**8.1**			0.03 µg/L	8.1
**PCB28***	**9.3**			0.05 µg/L	9.3
**PCB52***	**0.0**			0.3 µg/L	0.0
**PCB99**	**96.9**	0.04 µg/L	100.0	0.03 µg/L	95.0
**PCB101**	**53.6**	0.04 µg/L	75.5	0.03 µg/L	42.4
**PCB105**	**92.1**	0.04 µg/L	98.1	0.01 µg/L	88.9
**PCB128***	**49.4**			0.01 µg/L	49.4
**PCB138**	**100.0**	0.04 µg/L	100.0	0.01 µg/L	100.0
**PCB153**	**100.0**	0.04 µg/L	100.0	0.01 µg/L	100.0
**PCB156**	**98.0**	0.04 µg/L	100.0	0.01 µg/L	97.0
**PCB170**	**100.0**	0.04 µg/L	100.0	0.01 µg/L	100.0
**PCB180**	**100.0**	0.04 µg/L	100.0	0.01 µg/L	100.0
**PCB183**	**98.0**	0.04 µg/L	100.0	0.01 µg/L	97.0
**PCB187**	**100.0**	0.04 µg/L	100.0	0.01 µg/L	100.0

DL: Detection limit, * only measured in the 2011-2014 samples.
