# Supplementary material for: Serum levels of environmental pollutants is a risk factor for breast cancer in Inuit: a case control study
Source: Environ Health. 2017 Jun 13;16:56. doi: 10.1186/s12940-017-0269-6 (PMC5470290; doi:10.1186/s12940-017-0269-6)
Supplement: Supplementary file 3 — Odds ratio of breast cancer risk associated with PFAA stratified for recruitment period. (PDF 102 kb) [file 12940_2017_269_MOESM3_ESM.pdf]

Additional file 3

Serum levels of environmental pollutants is a risk factor for breast cancer in Inuit: a case control study

Maria Wielsøe, Peder Kern and Eva C. Bonefeld-Jørgensen

Odds ratio of breast cancer associated with PFAA stratified for recruitment period

		**Unadjusted analysis**			**Adjusted analysis**		
**Compounds**	**Collection**	**N (controls/cases)**	**OR (95% CI)**	***p***	**N (controls/cases)**	**OR (95% CI)**	***p***
∑PFCA	2000-2003	28/31	1.064 (0.975; 1.160)	0.162	28/31	1.067 (0.964; 1.181)	0.212
	2011-2014	53/46	1.025 (0.994; 1.058)	0.118	53/46	1.025 (0.994; 1.058)	0.118
∑PFSA	2000-2003	28/31	1.017 (1.002; 1.032)	**0.029**	28/31	1.017 (1.002; 1.032)	**0.029**
	2011-2014	53/46	1.012 (0.998; 1.025)	0.085	53/46	1.012 (0.998; 1.025)	0.085
∑PFAA	2000-2003	28/19	1.014 (1.001; 1.027)	**0.036**	28/19	1.014 (1.001; 1.027)	**0.036**
	2011-2014	53/46	1.008 (0.999; 1.017)	0.091	53/46	1.008 (0.999; 1.017)	0.091
PFHpA	2000-2003	-	-	-	-	-	-
	2011-2014	53/46	8.465 (0.902; 79.424)	0.061	40/35	1.860 (0.141; 24.577)	0.638
PFOA	2000-2003	28/31	1.244 (0.914; 1.693)	0.164	28/13	1.547 (0.945; 2.534)	0.083
	2011-2014	53/46	1.268 (0.911; 1.764)	0.160	44/35	0.823 (0.462; 1.464)	0.507
PFNA	2000-2003	28/31	1.208 (0.856; 1.705)	0.283	19/16	1.434 (0.834; 2.467)	0.193
	2011-2014	53/46	1.083 (0.985; 1.190)	0.100	53/46	1.026 (0.927; 1.135)	0.620
PFDA	2000-2003	28/31	1.502 (0.858; 2.629)	0.155	19/16	1.743 (0.694; 4.379)	0.237
	2011-2014	53/46	1.173 (0.960; 1.434)	0.119	53/46	1.031 (0.816; 1.302)	0.799
PFUnA	2000-2003	28/31	1.199 (0.907; 1.584)	0.203	19/16	1.333 (0.814; 2.183)	0.253
	2011-2014	53/46	1.056 (0.964; 1.156)	0.241	53/46	0.990 (0.892; 1.099)	0.852
PFDoA	2000-2003	28/31	2.771 (0.188; 40.898)	0.458	19/10	129.1 (0.38; 44135.8)	0.102
	2011-2014	53/46	1.189 (0.819; 1.726)	0.363	45/44	1.074 (0.642; 1.796)	0.786
PFHxS	2000-2003	28/31	1.269 (1.000; 1.610)	**0.050**	28/31	1.269 (1.000; 1.610)	**0.050**
	2011-2014	53/46	1.102 (0.950; 1.277)	0.200	53/46	1.014 (0.869; 1.184)	0.858
PFHpS	2000-2003	-	-	-	-	-	-
	2011-2014	53/46	1.360 (0.856; 2.160)	0.193			
PFOS	2000-2003	28/31	1.018 (1.002; 1.034)	**0.030**	28/31	1.018 (1.002; 1.034)	**0.030**
	2011-2014	53/46	1.013 (0.999; 1.027)	0.079	53/46	1.013 (0.999; 1.027)	0.079

n: number of observations per group; OR: odds ratio; 95% CI: 95% confidence interval; Unadjusted analysis: unadjusted estimates were reported; Adjusted: adjusted for confounders identified by change in estimate, following confounders were considered age, BMI, cotinine levels, parity, and

breastfeeding; **Bold** text: Significant finding; ∑PFCA: PFHpA, PFOA, PFNA, PFDA, PFUnA, PFDoA and PFTrA; ∑PFSA: PFHxS, PFOS and PFOSA; ∑PFAA: ∑PFCA + ∑PFSA.
