# Supplementary material for: Serum levels of environmental pollutants is a risk factor for breast cancer in Inuit: a case control study
Source: Environ Health. 2017 Jun 13;16:56. doi: 10.1186/s12940-017-0269-6 (PMC5470290; doi:10.1186/s12940-017-0269-6)
Supplement: Supplementary file 2 — Odds ratio of breast cancer risk associated with lipophilic POPs stratified for recruitment period. (PDF 133 kb) [file 12940_2017_269_MOESM2_ESM.pdf]

Additional file 2

Serum levels of environmental pollutants is a risk factor for breast cancer in Inuit: A case control study

Maria Wielsøe, Peder Kern and Eva C. Bonefeld-Jørgensen

Odds ratio of breast cancer risk associated with lipophilic POPs stratified for recruitment period

		Unadjusted analysis			Adjusted analysis		
**Compounds**	**Collection**	**N (controls/cases)**	**OR (95% CI)**	***p***	**N (controls/cases)**	**OR (95% CI)**	***p***
∑PCB	2000-2003	31/30	1.000 (1.000; 1.000)	0.422	31/30	1.000 (1.000; 1.000)	0.422
	2011-2014	53/46	1.000 (1.000; 1.001)	**0.012**	53/46	1.000 (1.000; 1.001)	**0.012**
∑PCB Grp1*	2000-2003	31/30	1.003 (0.999; 1.007)	0.141	31/30	1.003 (0.999; 1.007)	0.141
	2011-2014	53/46	1.004 (1.001; 1.008)	**0.012**	53/46	1.004 (1.001; 1.008)	**0.012**
∑PCB Grp2*	2000-2003	31/30	1.000 (0.999; 1.001)	0.982	31/30	1.000 (0.999; 1.001)	0.982
	2011-2014	53/46	1.001 (1.000; 1.002)	**0.009**	53/46	1.001 (1.000; 1.002)	**0.009**
∑PCB Grp3*	2000-2003	31/30	1.000 (1.000; 1.001)	0.234	31/30	1.000 (1.000; 1.001)	0.234
	2011-2014	53/46	1.000 (1.000; 1.001)	**0.016**	53/46	1.000 (1.000; 1.001)	**0.016**
∑DL-PCB	2000-2003	31/30	1.000 (0.996; 1.004)	0.992	31/30	1.000 (0.996; 1.004)	0.992
	2011-2014	53/46	1.005 (1.001; 1.010)	**0.011**	53/46	1.005 (1.001; 1.010)	**0.011**
∑OCP	2000-2003	31/30	1.000 (1.000; 1.001)	0.077	31/30	1.000 (1.000; 1.001)	0.077
	2011-2014	53/46	1.000 (1.000; 1.001)	**0.011**	53/46	1.000 (1.000; 1.001)	**0.011**
PCB99	2000-2003	31/30	1.003 (0.994; 1.012)	0.512	31/30	1.003 (0.994; 1.012)	0.512
	2011-2014	53/46	1.011 (1.001; 1.020)	**0.028**	53/46	1.011 (1.001; 1.020)	**0.028**
PCB101	2000-2003	31/30	0.976 (0.917; 1.039)	0.444	31/30	0.976 (0.917; 1.039)	0.444
	2011-2014	53/46	1.046 (0.962; 1.138)	0.292	53/46	1.046 (0.962; 1.138)	0.292
PCB105	2000-2003	31/30	1.000 (0.969; 1.031)	0.978	31/30	0.999 (0.964; 1.035)	0.948
	2011-2014	53/46	1.044 (0.996; 1.091)	**0.054**	53/46	1.044 (0.996; 1.091)	**0.054**
PCB118	2000-2003	31/30	1.003 (0.997; 1.009)	0.374	31/30	1.003 (0.997; 1.009)	0.374
	2011-2014	53/46	1.008 (1.001; 1.015)	**0.025**	53/46	1.008 (1.001; 1.015)	**0.025**
PCB138	2000-2003	31/30	1.000 (0.998; 1.002)	0.858	31/30	1.000 (0.998; 1.002)	0.858
	2011-2014	53/46	1.003 (1.001; 1.005)	**0.008**	53/46	1.003 (1.001; 1.005)	**0.008**
PCB153	2000-2003	31/30	1.001 (1.000; 1.002)	0.190	31/30	1.001 (1.000; 1.002)	0.190

	2011-2014	53/46	1.001 (1.000; 1.002)	**0.014**	53/46	1.001 (1.000; 1.002)	**0.014**
PCB156	2000-2003	31/30	0.991 (0.980; 1.003)	0.126	31/30	0.991 (0.980; 1.003)	0.126
	2011-2014	53/46	1.016 (1.004; 1.029)	**0.011**	53/46	1.016 (1.004; 1.029)	**0.011**
PCB170	2000-2003	31/30	1.001 (0.996; 1.005)	0.806	31/30	1.001 (0.996; 1.005)	0.806
	2011-2014	53/46	1.004 (1.001; 1.007)	**0.021**	53/46	1.004 (1.001; 1.007)	**0.021**
PCB180	2000-2003	31/30	1.001 (0.999; 1.002)	0.290	31/30	1.001 (0.999; 1.002)	0.290
	2011-2014	53/46	1.001 (1.000; 1.002)	**0.026**	53/46	1.001 (1.000; 1.002)	**0.026**
PCB183	2000-2003	31/30	1.008 (0.989; 1.027)	0.401	31/30	1.008 (0.989; 1.027)	0.401
	2011-2014	53/46	1.023 (1.005; 1.041)	**0.014**	53/46	1.023 (1.005; 1.041)	**0.014**
PCB187	2000-2003	31/30	1.002 (0.998; 1.006)	0.299	31/30	1.002 (0.998; 1.006)	0.299
	2011-2014	53/46	1.004 (1.001; 1.008)	**0.012**	53/46	1.004 (1.001; 1.008)	**0.012**
cis-Nonachlor	2000-2003	31/30	1.004 (0.995; 1.014)	0.337	31/30	1.004 (0.995; 1.014)	0.337
	2011-2014	53/46	1.010 (1.002; 1.018)	**0.011**	53/46	1.010 (1.002; 1.018)	**0.011**
Trans-Nonachlor	2000-2003	31/30	1.001 (0.999; 1.003)	0.186	31/30	1.001 (0.999; 1.003)	0.186
	2011-2014	53/46	1.002 (1.000; 1.003)	**0.007**	53/46	1.002 (1.000; 1.003)	**0.007**
HCB	2000-2003	31/30	1.000 (0.997; 1.003)	0.830	31/30	1.000 (0.997; 1.003)	0.830
	2011-2014	53/46	1.004 (1.001; 1.007)	**0.017**	53/46	1.004 (1.001; 1.007)	**0.017**
Mirex	2000-2003	31/30	1.001 (0.989; 1.014)	0.823	31/30	1.001 (0.989; 1.014)	0.823
	2011-2014	53/46	1.012 (1.001; 1.023)	**0.029**	53/46	1.012 (1.001; 1.023)	**0.029**
Oxychlordane	2000-2003	31/30	1.002 (0.999; 1.005)	0.184	31/30	1.002 (0.999; 1.005)	0.184
	2011-2014	53/46	1.002 (1.000; 1.004)	**0.014**	53/46	1.002 (1.000; 1.004)	**0.014**
p,p’DDE	2000-2003	31/30	1.001 (1.000; 1.001)	**0.036**	31/30	1.001 (1.000; 1.001)	**0.036**
	2011-2014	53/46	1.001 (1.000; 1.001)	**0.026**	53/46	1.001 (1.000; 1.001)	**0.026**
p,p’DDT	2000-2003	31/30	1.013 (0.992; 1.034)	0.233	31/30	1.013 (0.992; 1.034)	0.233
	2011-2014	53/45	1.017 (0.992; 1.042)	0.185	53/45	1.017 (0.992; 1.042)	0.185
βHCH	2000-2003	31/30	1.003 (0.981; 1.025)	0.795	31/30	1.003 (0.981; 1.025)	0.795
	2011-2014	53/46	1.022 (1.001; 1.044)	**0.042**	53/46	1.022 (1.001; 1.044)	**0.042**

n: number of observations per group; OR: odds ratio; 95% CI: 95% confidence interval; Unadjusted analysis: unadjusted estimates were reported; Adjusted: adjusted for confounders identified by change in estimate, following confounders were considered age, BMI, cotinine levels, parity, and breastfeeding; **Bold** text: Significant finding; ∑PCB: PCB 99, 101, 105, 118, 128, 138, 153, 156, 170, 180, 183, 187; ∑PCB group 1 (estrogenic PCBs): PCB 101, 187; ∑PCB group 2 (anti-estrogenic and dioxin-like PCBs): PCB 105, 118, 128, 138, 156, 170; ∑PCB group 3 (CYP1A1 and CYP2B inducing PCBs): PCB 99, 153, 180, 183; ∑DL-PCBs (dioxin-like PCBs): PCB 105, 118, 156; ∑OCP: p,p′-DDT, p,p′-DDE, mirex, β-HCH, HCB, cis- and trans-nonachlor, and oxychlordane.
